# COMPARISON OF PLASMA MALONDIALDEHYDE, GLUTATHIONE, GLUTATHIONE PEROXIDASE, HYDROXYPROLINE AND SELENIUM LEVELS IN PATIENTS WITH VITILIGO AND HEALTHY CONTROLS

**DOI:** 10.4103/0019-5154.39577

**Published:** 2008

**Authors:** I. Cetin Ozturk, Kadir Batcioglu, Fikret Karatas, Ersoy Hazneci, Metin Genc

**Affiliations:** *From Department of Biochemistry, Inonu University, Faculty of Medicine, Malatya/Turkey*; 1 *From Department of Biochemistry, Inonu University, Faculty of Pharmacy, Malatya/Turkey*; 2 *From Department of Chemistry, Firat University, Faculty of Science, Elazig/Turkey*; 3 *From Department of Dermatology, Acibadem Hospital, Bursa/Turkey*; 4 *From Inonu University, Faculty of Medicine, Public Health Department, Malatya/Turkey*

**Keywords:** *Free radicals*, *oxidative damage*, *selenium*, *vitiligo*

## Abstract

**Background:**

The etiology and pathophysiologic mechanism of vitiligo are still unclear. The relationship between increased oxidative stress due to the accumulation of radicals and reactive oxygen species and the associated changes in blood and epidermal component of vitiliginous skin have been reported many times. We investigated the possible changes of plasma malondialdehyde, glutathione, selenium, hydroxyproline and glutathione peroxidase activity levels in patients with vitiligo in order to evaluate the relationship between oxidative stress and etiopathogenesis of vitiligo.

**Materials and Methods:**

Plasma malondialdehyde, glutathione, hydroxyproline and glutathione peroxidase activity levels were measured by spectrophotometric methods, and HPLC was used for measurement of selenium concentrations.

**Results:**

Our results showed increased malondialdehyde, hydroxyproline and glutathione peroxidase activity levels in plasma of vitiligo group (*P* < 0.05).

**Conclusion:**

Support of antioxidant system via nonenzymatic antioxidant compounds and antioxidant enzymes may be useful to prevent of melanocyte degeneration which occur due to oxidative damage in vitiligo.

## Introduction

Vitiligo is an acquired, idiopathic, and in the majority of cases, a progressive, unpredictable disorder of the skin. The family history is positive in approximately 30-40% of cases, and there is no gender or racial bias.^1^ The onset is mostly early in life, and it has an estimated worldwide incidence of 0.5-4%. The etiology and pathogenic mechanism of vitiligo is still unclear. Several hypotheses have been proposed for the loss of functioning melanocytes in the skin of these patients. These include the presence of autoantibodies against various tissues; cytotoxic T-cells; autodestruction of melanocytes by intermediates of the melanogenesis pathway; the presence of intrinsic/extrinsic metabolic defects in the melanocytes themselves or in the epidermal melanin unit, leading to oxidative stress; and the neural hypothesis. Recently, a “convergence theory” has been suggested, combining all hypotheses of this disease.^1^,^2^ The role of free radicals and oxidative damage in the pathophysiology of vitiligo has been shown.^3^,^4^ The relationship between increased oxidative stress due to the accumulation of radicals and reactive oxygen species (ROS) and associated changes in blood and epidermal component of vitiliginous skin have been reported many times.^4^–^7^ An increased epidermal H_2_O_2_ level has been described both *in vivo* and *in vitro* in the active phase of vitiligo. High H_2_O_2_ level is possibly associated with reduced catalase, glutathione peroxidase activities and selenium levels.^3^,^8^

The tripeptide glutathione (GSH) is essential for metabolic and cell-cycle related functions in virtually all cells. Its ability to directly scavenge free radicals and to act as a co-substrate in the glutathione peroxidase catalyzed reduction of H_2_O_2_ and lipid hydroperoxides makes GSH central to defense mechanisms against intra- and extracellular oxidative stres.^9^

Malondialdehyde (MDA), the end product of lipid peroxidation (LPO), arising from the free radical degradation of polyunsaturated fatty acids, can cause cross-linking in lipids, proteins and nucleic acids.^10^,^11^ Human body is equipped with various antioxidants like superoxide dismutase (SOD), glutathione peroxidase (GSHPx) and catalase (CAT) which can counteract the deleterious action of ROS and protect from cellular and molecular damage.^12^,^13^ Hydroxyproline (HyPro) is a product of nonenzymatic hydroxylation reaction of proline. This nonezymatic hydroxylation reaction is caused by ROS. Collagen damage occurs in vitiligo. The degradation of collagen in vitiligo is possibly caused by ROS. Hydroxyproline is also a biochemical indice of collagen damage. This is why we determined the levels of Hydroxyproline in vitiligo. Hence we measured Hydroxyproline levels in blood samples of patients with vitiligo.

In this study, we investigated the possible changes of plasma MDA, GSH, GSSG, Se, hydroxyproline and glutathione peroxidase activity levels in patients with vitiligo in order to evaluate the relationship between oxidative stress and etiopathogenesis of vitiligo.

## Materials and Methods

### Experiment groups

Thirty (19 male, 11 female) patients with generalized stable vitiligo, and thirty (12 male, 18 female) healthy controls were included in this study. The ages of the patients ranged from 16 to 43 years old (23.6 ± 7.4). The control group consisted of healthy volunteers, whose ages ranged from 16 to 52 (27.9 ± 7.1). The Ethics Committee approved the protocol for this study; all subjects gave his/her informed consent. They were not under a therapeutic regimen for the previous 2 months and had not received drugs containing iron and/or vitamins. All individuals with any history of smoking and alcohol habits were excluded.

### Chemicals

All chemicals were purchased from Sigma Chemical Company (St. Louis, MO, USA).

### Preparation of plasma samples

All blood samples were drawn at the same time. Ten ml blood was drawn from median cubital vein of the patients and control group into tubes that were washed with heparin. The blood samples were centrifuged at 1000 × *g* for 10 min at +4°C, and upper plasma phase was drawn with pipette and transferred into polypropylene tubes, and stored at −40°C.

### Determination of protein levels

Protein determinations in plasma were done according to modified micro method of Lowry *et al.*,^14^ using BSA as standard.

### Estimation of glutathione peroxidase enzyme activity

GSHPx activity measurements were conducted according to Lawrence and Burk.^15^ 900 μL of 50 mM PBS solution (pH 7.4), including 5 mM EDTA, 2 mM NADPH, 20 mM GSH, 10 mM NaN_3_ and 23 mU of GSSG reductase were incubated at 37°C for 5 min. Fifty μL of 0.25 mM H_2_O_2_ solution and 50 µL of samples were added to the assay mixture. The change in absorbance at 340 nm was monitored for 3 min. A blank with all ingredients except plasma sample was also monitored. Specific activity was calculated as U/mg protein for plasma samples.

### Measurement of MDA levels

Lipid peroxidation products were quantified by the thiobarbituric acid (TBA) method.^16^ Malondialdehyde is formed as an end product of lipid peroxidation which reacts with TBA reagent under acidic conditions to generate a pink-colored product. Plasma (0.5 ml) was made up to 1 ml with saline and an equal volume of trichloroacetic acid (TCA) was added and incubated at 378C for 20 min and centrifuged at 500 *g*. To 1 ml of TCA extract (the supernatant) 0.25 ml TBA was added and heated in a water bath at 958C for 1 h till a faint pink color appeared. After cooling, the color was extracted in 1 ml butanol and the intensity was read at 532 nm. 1,1,3,3 tetra ethoxypropane (1-100 nmol/ml) was used as the standard.

### Measurement of glutathione levels

#### Total glutathione:

Glutathione was assayed according to modified Owens^17^ method. Plasma was first treated with metaphosphoric acid and centrifuged at 4000 g to obtain deproteinized samples. Then, supernatants were mixed with 5,5'-dithiobis-2-nitrobenzoat, NADPH_2_ and oxidized glutathione reductase in a sodium potassium phosphate buffer. The samples were incubated in a water bath at 30°C for 15 min, and absorbance was read spectrophotometrically at 412 nm.

#### Oxidized glutathione:

At first supernatants were treated with 2-vinylpyridine to prevent interference by any present reduced glutathione. Then, each sample was mixed with high concentrations of NADPH_2_ and GSSG reductase in the sodium potassium phosphate buffer. The change in absorbance was observed spectrophotometrically at 340 nm. When all the GSSG was run out, a constant absorbance was recorded.

### Measurement of selenium levels

Plasma samples were digested in a teflon bomb according to Breyer and Gilbert.^18^ Each sample was diluted with (v/v:1/3) 1.00 N HNO_3_/1.00 N HClO_4_ and left in the teflon bomb at 120°C for 12 h. After cooling to room temperature, Se^6+^ was reduced to Se^4+^ by addition of HCl to a final concentration of 4N and the samples kept at 90°C in a water-bath for 15 min. After returning to the room temperature, 5 ml of 0.1 M EDTA was added to each sample and mixed thoroughly. Two ml of 2.5 M HCOOH and 2 ml of 1500 ppm 3,3-diaminobenzidine (DAB) were added to each sample and the mixtures were left in a dark place for 60 min. The pH was adjusted to 7.0 with 4N NH_3_ and the Se-DAB complex was extracted with 5 ml of of toluene. These samples were analysed according to Watkinson^19^ and Whetter and Ullrey^20^ using a Perkin Elmer 100 fluorescence spectrophotometer with excitation at 420 nm and emission at 570 nm.

### Measurment of hydroxyproline levels

Hydroxyproline (HyPro) levels were measured by the method described by Bergman and Loxley.^21^ One mililiter of plasma samples were sealed in small Pyrex test tubes and hydrolyzed for 12 hours at 110°C by adding 5 mL of 6N HCl. The hydrolysates were neutralized by 2.5 N NaOH and 0.25 mL was used for analyses. Hydroxyproline oxidation was initiated by adding chloramine T and the tube contents were kept at room temperature for 5 min. Chloramine T was then removed by adding 3.15 M perchloric acid. After 5 min, 3.25 mL of Ehrlich's reagent was finally added; the mixture was shaken, and the tubes were placed in a 60°C water bath for 25 min. They were then cooled in tap water for 5 min. The absorbance values of the solutions were determined at 558 nm. The hydroxyproline values were calculated from L-hydroxyproline standard curve.

## Results and Discussion

Results are tabulated in Table 1. All results were expressed as mean ± SD.

**Table 1:** Activity of GSHPx and MDA, GSH, GSSG, Se and HyPro levels in vitiligo and control samples

	MDA (nmol/mL)	GSHPx (U/mg prot)	Se (ppb)	GSH (nmol/mL)	GSSG (nmol/mL)	HyPro (mg/L)
Vitiligo (*n* = 30)	0.642 ± 0.110^a^	0.550 ± 0.077^a^	122.333 ± 30.173	4.497 ± 0.486	4.45 × 10^2^− ± 0.6 × 10^2−^	16.841 ± 1.856^a^
Control (*n* = 30)	0.494 ± 0.085	0.439 ± 0.075	120.766 ± 21.802	4.567 ± 0.497	4.66 × 10^2−^ ± 0.55 × 10^2−^	15.013 ± 2.231

a *P* < 0.05, vitiligo group was compared to control group

The skin is the largest tissue of the human body with an approximate size of 1.8 m^2^, where numerous fine-tuned mechanisms act in a concerted action to keep the homeostasis in place. Recently, it has been shown *in vivo* and *in vitro* that patients with the pigmentation disorder vitiligo accumulate mM levels of hydrogen peroxide (H_2_O_2_) in their epidermis.^22^ It is well established that as high as millimolar levels of hydrogen peroxide lead to the inactivation of catalase and glutathione peroxidase despite normal mRNA expression.^3^,^23^,^24^ Possible sources of endogenous H_2_O_2_ production are increased activities of epidermal monoamine oxidase A, NADPH-oxidases, inducible nitric oxide synthase, increased levels of TNFα and photo-oxidation of epidermal 6-biopterin and sepiapterin.^25^ Increased generation or decreased removal of hydrogen peroxide has been shown to lead to lipid peroxidation, protein oxidation and DNA damage in skin and blood. H_2_O_2_ itself is a rather weak oxidizing agent incapable to induce these effect directly. It is generally believed that free metal ions, mostly Cu and Fe, are involved in the transmission of H_2_O_2_ induced cell toxicity by Fenton-like reactions.^26^ H_2_O_2_ has a longer half-time than other reactive oxygene molecules, singlet oxygen, superoxide or hydroxyl radicals and may diffuse to other compartments of body from place of generation. We reported at our preceding study that the erythrocyte glutathione peroxidase, catalase and Cu/Zn superoxide dismutase activities and plasma nitrite/nitrate levels were changed in patients with vitiligo.^27^ Wu SC *et al.*^28^ reported an increased skin blood flow and skin temperature in vitiligonous lesions. At these conditions, increased shear stress may be the cause of free radical production in blood.

In our study, we found increased MDA, HyPro and GSHPx activity levels in plasma of vitiligo group. Catalase and glutathione peroxidase catalyze the decomposition of hydrogen peroxide. Cohen and Hochstein,^29^ using the H_2_O_2_ diffusion technique, offered evidence that catalase does not play an important role in protecting blood cells against endogenous H_2_O_2_. Rather, they concluded glutathione peroxidase is the major route for disposing of H_2_O_2_. We know that concentration of H_2_O_2_ is high in vitiligonous area, and the diffusion of H_2_O_2_, into plasma will be relatively more limited (H_2_O_2_ which is thought to be in high concentration in lesion area) and significantly high GSHPx activities will be able to lead to the detoxification of H_2_O_2_ easily in the plasma. Ines *et al.*^30^ reported decreased GSHPx activities of erythrocytes in vitiligo patients. One of the sources of plasma antioxidant enzymes is lysis of erythrocytes. Increased plasma GSHPx activities may be the result of lysis of erythrocytes due to decreased GSHPx activity and increased ROS levels in erythrocytes. On the other hand, as the activity of GSHPx is high in plasma, it led us to consider those antioxidant enzymes or molecules that may lead to synthesis of mRNA for antioxidants that are likely to be transported to blood from the other components of body.

The consequence of increased free radicals via H_2_O_2_ generation and imbalances in oxidant/antioxidant balance is oxidative stress, which leads to oxidative damage, resulting in increased MDA levels, which is the end product of lipid peroxidation. Koca *et al.*^31^ has reported increased serum MDA levels in vitiligo. Similar findings have been reported by Ines *et al.*^30^ and Yildirim *et al.*^32^. Our results support the findings of these articles. On the other hand, we found increased plasma HyPro levels in vitiligo patients. Possible reasons of high HyPro levels may be the damage of collagen tissue and nonenzymatic oxidation of proline via free radicals. There is no study that evaluates the relationship HyPro levels and vitiligo. However, it is known that both of free and constitutional amino acids may be oxidized by free radicals.

In our study we observed a slight but not significant increase in plasma Se levels. Beazley *et al.*^24^ reported increased serum Se levels in vitiligo. Similar results were reported by Garsaud *et al.*^33^. Recently, Picardo *et al.*^34^ have claimed that a cocktail containing various antioxidants, including selenomethionine, is beneficial in the repigmentation process in vitiligo. Selenium, which is a crucial factor for the function of the selenium-dependent isoenzyme of glutathione peroxidase, has been shown to be elevated in the serum of patients with vitiligo by other authors.^24^,^35^,^36^ In addition, our results showed that plasma GSH and GSSG levels did not change significantly. GSH is a component of nonenzymatic antioxidant system and substrate for GSHPx. Passi *et al.*^37^ showed that the epidermal GSH levels of active vitiligo patients were significantly lower when compared with the controls. Park *et al.*^38^ reported that glutathione prevented dopamine-induced apoptosis of melanocytes.

In conclusion, vitiligo affects not only skin but also other components of organism such as blood. However, the effects on plasma is lower than skin. In addition, we believe that the support of antioxidant system via nonenzymatic antioxidant compounds and antioxidant enzymes may be useful in the prevention of melanocyte degeneration, which occurs due to oxidative damage in vitiligo.
